# The Emerging Role of N6-Methyladenosine RNA Methylation as Regulators in Cancer Therapy and Drug Resistance

**DOI:** 10.3389/fphar.2022.873030

**Published:** 2022-04-06

**Authors:** Zhaolin Chen, Ying Hu, Le Jin, Fan Yang, Haiwen Ding, Lei Zhang, Lili Li, Tingting Pan

**Affiliations:** ^1^ Department of Pharmacy, The First Affiliated Hospital of USTC, Division of Life Sciences and Medicine, University of Science and Technology of China, Anhui Provincial Hospital, Hefei, China; ^2^ Inflammation and Immune Mediated Diseases Laboratory of Anhui Province, School of Pharmacy, Anhui Medical University, Hefei, China; ^3^ Department of Clinical Medical, The First Clinical Medical College, Anhui Medical University, Hefei, China; ^4^ Department of Hematopathology, The First Affiliated Hospital of Anhui Medical University, Hefei, China; ^5^ Department of General Surgery, Diagnosis and Therapy Center of Thyroid and Breast, The First Affiliated Hospital of USTC, Division of Life Sciences and Medicine, University of Science and Technology of China, Anhui Provincial Hospital, Hefei, China

**Keywords:** N6-methyladenosine (m6A), RNA methylation, inflammation, cancer, therapeutic targets

## Abstract

N6-methyladenosine (m^6^A) RNA methylation has been considered the most prevalent, abundant, and conserved internal transcriptional modification throughout the eukaryotic mRNAs. Typically, m^6^A RNA methylation is catalyzed by the RNA methyltransferases (writers), is removed by its demethylases (erasers), and interacts with m^6^A-binding proteins (readers). Accumulating evidence shows that abnormal changes in the m^6^A levels of these regulators are increasingly associated with human tumorigenesis and drug resistance. However, the molecular mechanisms underlying m^6^A RNA methylation in tumor occurrence and development have not been comprehensively clarified. We reviewed the recent findings on biological regulation of m^6^A RNA methylation and summarized its potential therapeutic strategies in various human cancers.

## Introduction

Dynamic and reversible chemical modifications, especially methylation on DNA and histone proteins, are important for epigenetic control of gene expression (Wang et al., 2017). Recently, accumulating attention on the involvement of post-transcriptional RNA modifications in bioscience research has begun to be explored. To date, more than 100 distinct post-transcriptional chemical modifications have been identified in RNA among all living organisms. Several common types of RNA modifications include pseudouridine (Ψ), N1-methyladenosine (m^1^A), N6-methyladenosine (m^6^A), 5-methylcytosine (m^5^C), 1-methylguanosine (m^1^G), 2-methylguanosine (m^2^G), 6-methylguanosine (m^6^G), and 7-methylguanosine (m^7^G). In brief, m^1^G, m^2^G, and m^1^A modifications restrain the synthesis of proteins (Sun et al., 2019). Among these modifications, N6-methyladenosine (m^6^A), methylated at the N6 position of adenosine, discovered in the early 1970s, has been identified as the most prevalent and abundant mRNA modification in eukaryotic mRNAs (Desrosiers et al., 1974). Furthermore, with the application of advanced technologies including m^6^A sequencing (m^6^A-seq), methylated RNA immunoprecipitation sequencing (MeRIP-seq), and m^6^A-sensitive RNA-endoribonuclease-facilitated sequencing (m^6^A-REF-seq), m^6^A modifications sites have been detected existing in various types of RNA except only in mRNA, such as transfer RNAs (tRNAs), noncoding RNAs (ncRNAs), and small nucleolar RNAs (snRNAs) (Dominissini et al., 2012; Cui et al., 2016; Zhang et al., 2019). It has been shown that the abundance of m^6^A modifications is about 25% of transcripts. Primarily occurring in the consensus sequence RRACH (R: purine = A or G; A: m^6^A; H: non-guanine base = A, C, or U), m^6^A modifications are considerably enriched near stop codons, in 5′- and 3′-untranslated terminal regions (UTRs) and within long internal exons (Meyer et al., 2012). Analogous to the epigenetic regulation of DNA and histone methylations, m^6^A modifications are a dynamic and reversible process in mammals which are regulated by methyltransferase and demethylase and regulate the expression of post-transcriptional genes without changing the base sequence. However, the regulatory mechanisms of m^6^A are complex (Zhou et al., 2020a). Emerging evidences have explored that m^6^A plays a vital role in pre-mRNA splicing, 3′-end processing, translation regulation, nuclear export, mRNA decay, and ncRNA processing. These reversible processes are also needed for various aspects, including somatic cell reprogramming, embryonic stem cell differentiation, and progression in diversified diseases, by regulating the biological functions of cells (Yang et al., 2020a).

Recently, an increasing number of studies have reported that m^6^A RNA methylation performed its important and diverse biological functions in tumorigenesis and cancer progression (Huang et al., 2021a). In this review, we mainly provide an exhaustive summary of the biological functions of m^6^A modification as regulators in cancer therapy and drug resistance, in order to explore new diagnostic biomarkers and potential therapeutic targets.

## Regulators of m^6^A: m^6^A Writers, Erasers, and Readers

**FIGURE 1:**
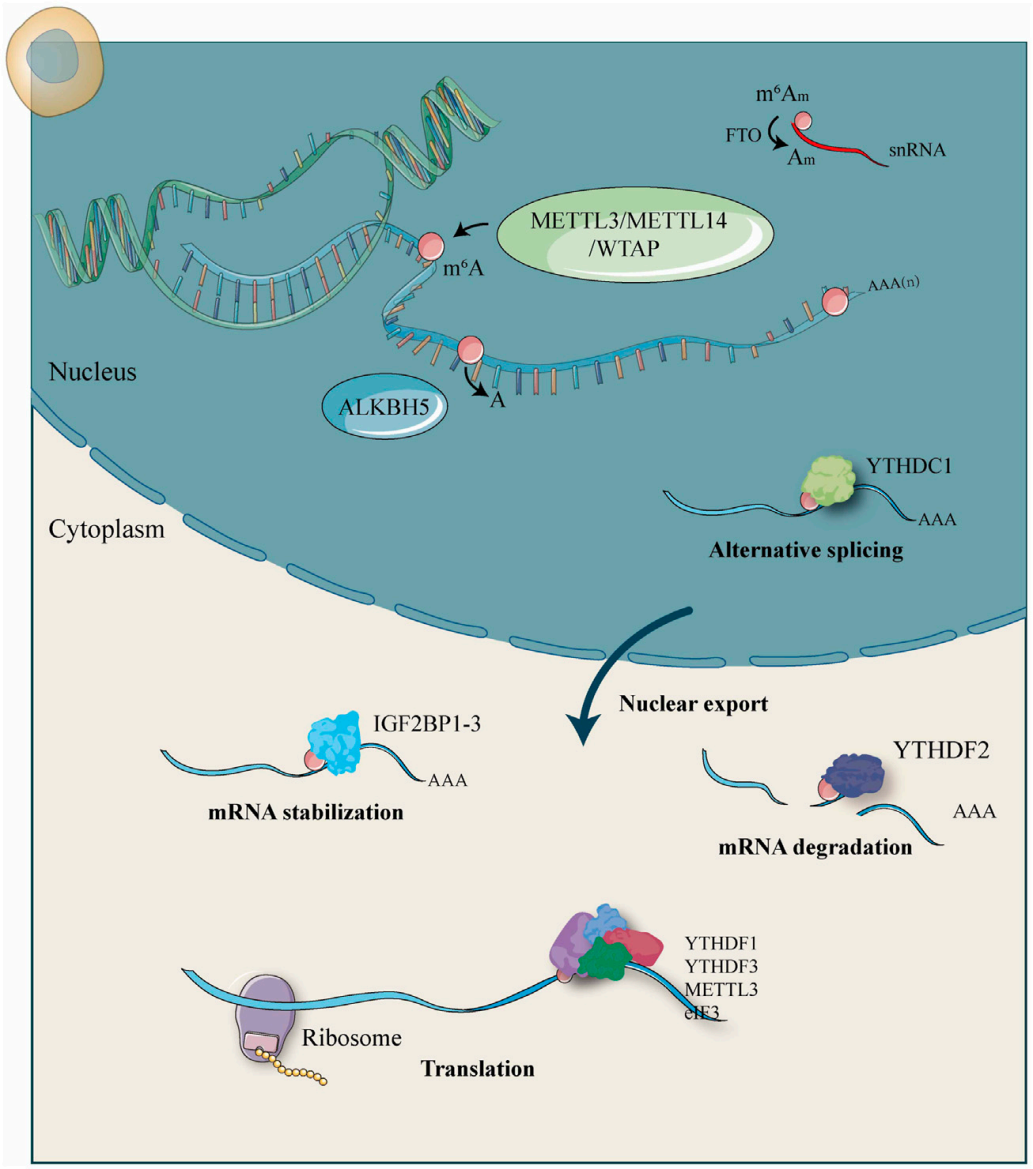
Molecular functions and mechanisms of m^6^A RNA methylation by m^6^A “writer,” “eraser,” and “reader” proteins.

RNA m^6^A modification occurs at the sixth N of RNA adenine (A) and is regulated by a large methyltransferase complex involving three homologous proteins identified as “writers,” “erasers,” and “readers” (Li et al., 2020a). These regulators have been shown to participate in RNA metabolic processes, such as alternative splicing, export, RNA stability, translation efficiency, or localization (Figure 1). Crosslink among m^6^A writers, erasers, and readers is involved in pathogenesis and disease progression of human cancers.

### m^6^A Writers

The first type of protein is the highly conserved mRNA methyltransferase complex (MTC) termed as “writers.” M^6^A modification is catalyzed co-transcriptionally through the MTC that consists of the METTL complex (MAC), namely a METTL3–METTL14 heterodimer core and their cofactors METTL-associated complexes (MACOM) such as WTAP, VIRMA (KIAA1429), RBM15, RBM15B, and ZC3H13 (Deng et al., 2018; Zaccara et al., 2019). In addition to the MTC, other writers have also been identified in recent years, including METTL5, METTL16, and ZCCHC4, which exhibit their regulation roles for the deposition of m^6^A into structured RNAs (Aoyama et al., 2020; Ignatova et al., 2020; Pinto et al., 2020). Found in 1997, METTL3 was initially isolated from HeLa cells, and it contained two S-adenosylmethionine binding sites which were called the catalytically active methyltransferase domain. METTL3 widely exists in eukaryotes and is highly conserved in mammals (Bokar et al., 1997). METTL3 usually forms a stable heterodimer with METTL14 at a ratio of 1:1, which is required to enhance enzymatic activity of METTL3 through a RNA-binding substrate and positioning the methyl group for transfer to adenosine. Due to the synergistic effect based on a physical connection, the heterodimer of METTL3–METTL14 exhibits enhanced methylation efficiency (Wang et al., 2016). In HeLa cells, knockdown of METTL3 or METTL14 reduced the total m^6^A level (Liu et al., 2014). In skin cancer, METTL14 knockdown decreased the m^6^A levels and UVB-induced cyclobutene pyrimidine dimer repair (Yang et al., 2021). Furthermore, research studies pointed out that WTAP as a methyltransferase ensures the stability and localization of the METTL3–METTL14 heterodimer into nuclear speckles, which enrich with pre-mRNA processing factors and promote catalytic activity of the heterodimer (Schöller et al., 2018). Notably, WTAP silencing resulted in the largest decrease of m^6^A levels, and thus, WTAP recruited METTL3 and METTL14 to their target mRNAs (Liu et al., 2014). Interestingly, VIRMA selectively promotes mRNA m^6^A methylation near 3′UTR and stop codon regions and guides region-selective methylations by recruiting the catalytic core METTL3–METTL14–WTAP complex (Yue et al., 2018). RBM15 and its paralogue RBM15B bind to the METTL3–METTL14 complex and recruit it to target transcripts that catalyze the m^6^A modification on mRNA (Patil et al., 2016). ZC3H13 is a canonical CCCH zinc-finger protein, in concert with other cofactors such as WTAP, which modulates RNA m^6^A methylation in the nucleus (Zhu et al., 2019a). Wen J *et al.* found that Zc3h13 down-regulation caused an obvious decrease of the m^6^A level on mRNA in mouse embryonic stem cells. Furthermore, ZC3H13 was shown to regulate nuclear RNA m^6^A methylation by the Zc3h13–WTAP–virilizer–Hakai complex (Wen et al., 2018). More recently, it was found that METTL16, a newly discovered independent RNA methyltransferase, can induce N6-methylation in the 3′-UTR of mRNAs and A43 of the U6 snRNA, playing a critical role in mRNA stability and splicing (Warda et al., 2017). Hiroki *et al.* reported that METTL16 and YTHDC1 are involved in MAT2A mRNA stabilization, which allows cells to monitor and maintain intracellular S-adenosylmethionine levels (Shima et al., 2017).

### m^6^A Demethylases (Erasers)

The m^6^A demethylase represented by FTO and ALKBH5 is the second type of protein involved in m^6^A regulation which can demethylate the N6-position of A from target mRNA in FeII/a-ketoglutarate-dependent dioxygenases manner; its coding gene is called “erasers” (Wang et al., 2020a; Wang et al., 2020b). FTO was found in a fusion toe mutant mouse and was shown to be the m^6^A mRNA demethylase in 2011 (Jia et al., 2011). Using transcriptome analyses and m^6^A-seq, it revealed that FTO regulates gene expression and mRNA splicing of grouped genes. FTO depletion mediates m^6^A modification, promotes the RNA binding ability of SRSF2 protein to target exonic splicing enhancers, and increases inclusion of target exon 6, thus inhibiting preadipocyte differentiation (Zhao et al., 2014). Silence of FTO increased, whereas overexpression of FTO decreased total m^6^A levels in mRNA in Hela and 293FT cells (Fu et al., 2013). Similarly, FTO deletion increased m^6^A RNA methylation and inhibited arsenic-induced tumorigenesis (Cui et al., 2021). However, works of research on the specific substrate of FTO have produced some contradictions. A study by Mauer *et al.* revealed that FTO preferentially demethylates N6, 2′-O-dimethyladenosine (m^6^A_m_) rather than m^6^A, and reduces the stability of m^6^A_m_ mRNAs. FTO knockout increased m^6^A_m_ levels without increasing m^6^A levels *in vitro* and *in vivo*, suggesting FTO targets m^6^A_m_. Therefore, the data showed that FTO is an m^6^A_m_ “eraser” and forms 2′-O-methyladenosine (A_m_) in cells (Mauer et al., 2017). This confusion was further elucidated by subsequent research. FTO mediates the demethylation of m^6^A and m^6^A_m_ with polyA-tailed RNA. FTO locates in the nucleus mediates the demethylation of m^6^A, and FTO in the cytoplasm removes the methyl group of m^6^A_m_ and m^6^A (Wei et al., 2018). To resolve these conflicting results, researchers recently have developed the m^6^A-Crosslinking-Exonuclease-sequencing (m^6^ACE-seq) method which can map transcriptome-wide m^6^A and m^6^A_m_ at quantitative single-base-resolution. Using m^6^ACE-seq on *Fto*-KO RNA and identifying 273 sites with relative methylation levels accumulations as FTO-regulated sites, the results showed that FTO loss causes disruptive m^6^A_m_ accumulation (Koh et al., 2019). ALKBH5 is another m^6^A demethylase that can selectively remove the methyl group from m^6^A rather than m^6^A_m_ in mRNA and other types of nuclear RNA (Panneerdoss et al., 2018). ALKBH5 protein has an alanine-rich sequence and a curly helix structure at its N-terminal, which plays an important role in its nuclear localization (Wang et al., 2020b). The depletion of ALKBH5 led to an increased m^6^A level, while its up-regulation in human cell lines resulted in a decrease of m^6^A modification on mRNA (Wu et al., 2018).

### m^6^A-Binding Proteins (Readers)

The genes encoding the third type of m^6^A regulatory proteins are known as “readers,” which recognize m^6^A, bind the RNA and initiate corresponding functions (Dai et al., 2021). The earliest readers were coding genes in the YT521-B homology (YTH) domain family proteins, including YTHDFs subtypes (YTHDF1, YTHDF2, and YTHDF3) located in the cytoplasm and YTHDCs subtypes (YTHDC1 and YTHDC2) in the nucleus, which can improve the efficiency of mRNA translation. Several m^6^A readers with YTH domain located in the cytoplasmic compartment (YTHDF1, YTHDF2, and YTHDF3) and nuclear compartment (YTHDC1) have been identified and possess differential functions based on their molecular features and cellular localization (Shi et al., 2021a). Subsequently, other readers were found, including IGF2BPs and HNRNPs. However, the biological functions of m^6^A modification remain unclear. It is worth noting that YTHDF protein subunits (YTHDF1/2/3) are similar in domain structures which all contain a *C*-terminal YTH domain and an *N*-terminal low complexity sequence but have different functions (Shi et al., 2019). Among these, YTHDF2 was the first identified and showed to bind to m^6^A located in 3′UTR and accelerate its target transcripts degradation by localizing m^6^A-modified mRNA to processing them in the cytosol (Du et al., 2016). On the contrary, cytoplasmic YTHDF1 and YTHDF3 promote target transcripts translation in HeLa cells through recruiting translation initiation factors (Wang et al., 2015; Shi et al., 2017). Several studies have reported that knockdown of YTHDF2 and YTHDF3 can lead to an obvious increase in m^6^A-modified mRNAs in cells (Shi et al., 2017; Zhang et al., 2020a).

In addition, YTHDC1 is a nuclear protein involved in pre-mRNA splicing (Chen et al., 2020a). Strikingly, YTHDC1 can regulate the alternative splicing of pre-mRNA by facilitating SRSF3 while blocking SRSF10 mRNA binding to nuclear speckles (Xiao et al., 2016). YTHDC2, as another m^6^A reader, selectively binds m^6^A at its consensus motif. YTHDC2 mediated mRNA stability and translation and particularly regulated spermatogenesis (Hsu et al., 2017). YTHDC2 knockdown inhibited the metastatic ability of tumor cells through a translation-dependent pathway (Tanabe et al., 2016). Moreover, distinct from YTH domain-containing proteins, a different class of readers has been shown to utilize common RNA binding domains (RBDs) to bind m^6^A-containing transcripts preferentially (Shi et al., 2019). Several IGF2BPs fall into this category, such as IGF2BP1/2/3, which recognize the consensus GG(m^6^A)C sequence and enhance the stability and storage of the target mRNAs in an m^6^A-dependent manner (Huang et al., 2018). However, it is not fully understood whether these proteins bind to m^6^A directly. Interesting, recent studies have mentioned that the HNRNP protein family can selectively bind to m^6^A-methylated transcripts through the m^6^A switch. Among these, HNRNPC and HNRNPG protein as nuclear m^6^A readers could affect the local secondary structure of mRNAs and lncRNAs (Liu et al., 2015). Another HNRNP member, HNRNPA2B1, selectively binds to GGAG or GGCU motifs on miRNA. Loss of HNRNPA2B1 caused a decrease in exosomal loading of GGAG-containing miRNAs in hepatocytes, showing that there is a specific class of miRNAs sorting into exosomes (Yang et al., 2020b). Surprisingly, the newer findings have challenged the idea that HNRNPA2B1 protein may bind an unfolded RNA due to m^6^A (Liu and Shi, 2021).

Generally, new writers, erasers, and readers related to m^6^A modification are still being identified, implying that further research is left to explore the potential regulation of biological functions of m^6^A modification.

## M^6^A Regulators-Guided Epigenetic Modification in Cancers

Currently, several studies have hinted that m^6^A modifications control RNA production/metabolism and are involved in human carcinogenesis. The multiple characteristics of m^6^A modifications and their related regulators take part in various cancers, such as leukemia, lung cancer, and hepatoma*.* M^6^A regulators could function as a tumor promoter or a tumor suppressor which regulate the expression of tumor oncogenes or anti-oncogene, thereby affecting cancer progressions (Table 1).

**TABLE 1:** Roles of aberrant m^6^A modification in various cancers.

Cancer type	M^6^A regulator	Target gene	Function	Regulation	Mechanism of m^6^A recognition	Reference
AML	METTL3	SP1	Oncogene	Up-regulation	Promote SP1 translation, promote cell proliferation and growth, and inhibit cell differentiation	29186125
	METTL14	MYB and MYC	Oncogene	Up-regulation	Stabilize MYB and MYC mRNA, increase MYB and MYC expressions, inhibit cell differentiation, and induce cell proliferation/survival	29290617
	WTAP	mTOR	Oncogene	Up-regulation	Increase the phosphorylation levels of mTOR, promote cell proliferation and colony formation, and inhibit differentiation	24413322
	FTO	ASB2 and RARA	Oncogene	Down-regulation	Destabilize ASB2 and RARA mRNA, decrease ASB2 and RARA expressions, suppress ATRA-induced cell differentiation, and enhance cell transformation and leukemogenesis	28017614
	FTO/YTHDF2	MYC and CEBPA	Oncogene	Up-regulation	Stabilize MYC and CEBPA mRNA, increase MYC and CEBPA expressions, and promote cell proliferation	29249359
	FTO	PFKP and LDHB	Oncogene	Up-regulation	Stabilize PFKP and LDHB mRNA, increase PFKP and LDHB expressions, and promote leukemogenesis	33434505
	ALKBH5	TACC3	Oncogene	Down-regulation	Destabilize TACC3 mRNA, decrease TACC3 expression, and promote cell transformation, development, and maintenance	32402250
	YTHDF2	Tal1	Anti-oncogene	Down-regulation	Destabilize Tal1 mRNA, decrease Tal1 expression, and decrease cell expansion	30065315
	YTHDF2	TNFR2	Oncogene	Down-regulation	Destabilize Tnfrsf1b mRNA, decrease Tnfrsf1b expression, and decrease TNF-induced apoptosis	31031138
	YTHDC1	MCM4	Oncogene	Up-regulation	Stabilize MCM4 mRNA, increase MCM4 expression, increase DNA replication, and promote leukemogenesis	34255814
	YTHDC1	MYC	Oncogene	Down-regulation	Stabilize MYC mRNA, increase MYC expression, and promote leukemogenesis	34048709
	IGF2BP1	ALDH1A1, HOXB4, and MYB	Oncogene	—	Decrease the expressions of ALDH1A1, HOXB4, and MYB, promote cell tumorigenicity, decrease myeloid differentiation, and induce chemotherapeutic drug resistance	31768017
LC	METTL3	Bcl-2	Oncogene	Up-regulation	Increase Bcl-2 expression and facilitate tumorigenesis	34132367
	METTL3	EZH2	Oncogene	Up-regulation	Increase EZH2 expression	32373962
	METTL3	JUNB	Oncogene	Up-regulation	Stabilize JUNB mRNA, increase JUNB expressions, and promote leukemogenesis	31982139
	METTL3	FBXW7	Anti-oncogene	Up-regulation	Increase FBXW7 translation and expression and suppress tumorigenesis	33676554
	FTO	MZF1	Oncogene	Down-regulation	Destabilize MZF1 mRNA, decrease MZF1 expression, and promote tumorigenesis	29842885
	FTO	USP7	Oncogene	Down-regulation	Destabilize USP7 mRNA, decrease USP7 expression, and promote tumorigenesis	30905413
	FTO	E2F1	Oncogene	Down-regulation	Decrease E2F1 expression and promote cell migration, invasion, and metastasis	34169146
	ALKBH5	YAP	Anti-oncogene	Down-regulation	Decrease YAP expression and inhibit tumor growth and metastasis	32106857
	ALKBH5	TIMP3	Oncogene	Down-regulation	Destabilize TIMP3 mRNA, decrease TIMP3 expression, and promote tumor progression	31927006
	ALKBH5/YTHDF2	SOX2, SMAD7, and MYC	Oncogene	—	Prevent decay of SOX2, SMAD7, and MYC mRNAs	34016959
	YTHDF2	6PGD	Oncogene	Up-regulation	Increase the 6PGD protein level by facilitating its mRNA translation and promote tumorigenesis	31504235
	YTHDC2	SLC7A11	Anti-oncogene	Up-regulation	Stabilize SLC7A11 mRNA, increase SLC7A11 expression, and inhibit tumorigenesis	33232910
	YTHDC2	HOXA13	Anti-oncogene	Up-regulation	Stabilize HOXA13 mRNA and increase HOXA13 expression	33785413
HCC	METTL3/YTHDF2	SOCS2	Oncogene	Down-regulation	Destabilize SOCS2 mRNA, decrease SOCS2 expression, promote cell proliferation, migration, colony formation, tumorigenicity, and lung metastasis	29171881
	METTL14	USP48	Anti-oncogene	Down-regulation	Destabilize USP48 mRNA, decrease USP48 expression, and inhibit cell proliferation, migration, and invasion	33903120
	METTL16	eIF3a/b	Oncogene	Up-regulation	Enhance the translation efficiency of eIF3a/b and promote cell proliferation, migration and invasion, and tumor growth	35145225
	WTAP	ETS1	Oncogene	Down-regulation	Destabilize ETS1 mRNA, decrease ETS1 expression, and promote the proliferation capability and tumor growth	31438961
	FTO	CUL4A	Anti-oncogene	Up-regulation	Increase CUL4A protein expression and inhibit cell proliferation in DEN-induced HCC mice	32956847
	FTO	PKM2	Oncogene	Up-regulation	Increase PKM2 expression, promote cell proliferation, and inhibit cell apoptosis	31632576
	ALKBH5/IGF2BP1	LYPD1	Anti-oncogene	Up-regulation	Stabilize LYPD1 mRNA, increase LYPD1 expression, and inhibit migration, invasion, and metastasis	32772918
	ALKBH5	HBx	Oncogene	Up-regulation	Stabilize HBx mRNA and promote the HBV-HCC cell growth and migration	34112124
	YTHDF2	OCT4	Oncogene	Up-regulation	Increase OCT4 protein expression and promote the liver CSC phenotype and cancer metastasis	32366907
	IGF2BP2	FEN1	Oncogene	Up-regulation	Stabilize FEN1 mRNA, increase FEN1 expression, and promote cell proliferation and tumor growth	33224879
GBM	METTL3/14	ADAM19	Anti-oncogene	Down-regulation	Decrease ADAM19 expression and inhibit tumorigenesis	28297667
	METTL3	SOX2	Oncogene	Up-regulation	Stabilize FEN1 mRNA, increase FEN1 expression, and promote cell proliferation, tumor growth, and radioresistance	28991227
	METTL3/YTHDC1	SRSF3/6/11	Oncogene	Up-regulation	Stabilize SRSFs mRNA, increase SRSFs expression, and promote cell proliferation and tumor growth	31530567
	METTL3/YTHDF1	ADAR1	Oncogene	Up-regulation	Increase ADAR1 protein expression and promote cell proliferation and tumor growth	33509238
	ALKBH5	FOXM1	Oncogene	Up-regulation	Stabilize FOXM1 mRNA and increase its expression by interacting with FOXM1-AS and promote tumorigenesis	28344040
	YTHDF2	MYC	Oncogene	Up-regulation	Stabilize MYC mRNA, increase MYC expression, and promote cell proliferation and tumor growth	33023892
BC	METTL3	HBXIP	Oncogene	Up-regulation	Increase HBXIP expression and promote cell proliferation and tumor growth	29174803
	METTL3	Bcl-2	Oncogene	Up-regulation	Increase Bcl-2 expression, promote cell proliferation, and inhibit apoptosis	31454538
	METTL3	COL3A1	Anti-oncogene	Up-regulation	Increase COL3A1 expression and suppress migration, invasion, and adhesion	32766145
	METTL14	CXCR4 and CYP1B1	Oncogene	Up-regulation	Stabilize CXCR4 and CYP1B1 mRNA, increase CXCR4 and CYP1B1 expressions, and promote cell proliferation and growth	32576970
	FTO	BNIP3	Oncogene	Down-regulation	Decrease BNIP3 expression and promote cell proliferation, tumor growth, and metastasis	30922314
	ALKBH5	NANOG	Oncogene	Up-regulation	Stabilize NANOG mRNA, increase NANOG expression, and promote BCSCs enrichment and tumor formation	27001847
CC	METTL3/YTHDF1	HK2	Oncogene	Up-regulation	Stabilize HK2 mRNA, increase HK2 expressions, and promote glycolysis and proliferation	33099572
	METTL3/IGF2BP3	RAB2B	Oncogene	Up-regulation	Stabilize RAB2B mRNA, increase RAB2B expression, and promote proliferation	32339511
	FTO	E2F1 and Myc	Oncogene	Up-regulation	Increase E2F1 and Myc expressions and promote proliferation and migration	31827395
	YTHDF1	RANBP2	Oncogene	Up-regulation	Increase RANBP2 protein expression, promote cell proliferation, migration, invasion, and tumor growth, and inhibit apoptosis	33816306
OC	METTL3	AXL	Oncogene	Up-regulation	Increase AXL protein expression, promote cell proliferation, migration, invasion, and tumor formation	30249526
	YTHDF1	EIF3C	Oncogene	Up-regulation	Stabilize EIF3C protein, increase EIF3C protein expression, and promote cell proliferation, migration, invasion, and metastasis	31996915
	YTHDF2	BMF	Oncogene	Down-regulation	Destabilize BMF mRNA, decrease BMF mRNA expression, and promote cell proliferation and growth	33658012

### Acute Myeloid Leukemia

Acute myeloid leukemia (AML) is one of the most common types of acute leukemia with distinct genetic and molecular abnormalities in adults. Despite advances in medical treatment, only a small proportion of AML patients can survive for over five years after diagnosis with the current standard chemotherapies (Döhner et al., 2017). Emerging evidence suggested that m^6^A RNA methylation is involved in biological processes, including cell differentiation, proliferation, apoptosis, therapeutic resistance, and LSCs/LICs self-renewal of AML. An independent research revealed that METTL3 is elevated in AML and binds to the SP1 promoter region with the assistance of transcription factor CEBPZ, facilitating SP1 translation *via* relieving ribosome stalling (Barbieri et al., 2017). Consistent with METTL3, down-regulation of METTL14 decreased the MYB and MYC expression and eventually induced myeloid differentiation of HSPCs, cell growth inhibition, and cell death of AML (Weng et al., 2018). Similar to METTL3 and METTL14, WTAP was up-regulated in AML patient samples and cell lines compared to normal mononuclear cells (Bansal et al., 2014). WTAP mRNA is m^6^A methylated and bound by cytoplasmic METTL3. METTL3 knockdown increases the mRNA and protein levels of WTAP. However, in the absence of a functional METTL3, WTAP up-regulation alone is not sufficient to increase cell proliferative growth in AML cells, astricting its oncogenic function to its involvement in the m^6^A methylation complex (Sorci et al., 2018).

In addition, FTO is overexpressed in AML patients carrying t(11q23)/MLL rearrangements, t(15; 17)/PML-RARA fusion, FLT3-ITD and/or NPM1 mutations. The study then showed that FTO decreases m^6^A levels on the UTRs of ASB2 and RARA through its eraser activity, thereby contributing to the response of AML cells to all-trans-retinoic acid treatment and leukemogenesis (Li et al., 2017). Interesting, a study carried out by Su *et al.* demonstrated that the R-2HG/FTO/m^6^A axis decreases the stability of MYC and CEBPA transcripts and thus inhibits downstream pro-tumor pathways in AML. On the other hand, YTHDF2 is associated with MYC and CEBPA to facilitate m^6^A modification in the 5′-UTR and CDS (Su et al., 2018). Recently, a new report has shown that R-2HG treatment or FTO inhibition abrogates m^6^A/YTHDF2-mediated post-transcriptional up-regulation of two critical glycolytic genes PFKP and LDHB expressions, thereby reducing aerobic glycolysis and playing a critical tumor-promoting role in the pathogenesis of AML (Qing et al., 2021). A previous study based on the analysis of the TCGA AML cohort dataset by Kwok *et al.* reported that ALKBH5 is markedly deleted in AML patients, especially in TP53 mutant cases (Kwok et al., 2017). However, Chen *et al.* has demonstrated that ALKBH5 levels are abnormally elevated in AML, which correlates with poor prognosis in AML patients. TACC3, as a direct and functionally important target of ALKBH5, is related to substantially decreased expression level and increased m^6^A abundance upon knockdown of ALKBH5. Strikingly, ALKBH5 regulates TACC3 expression more likely by influencing TACC3 mRNA stability instead of translation (Shen et al., 2020).

Li and his colleagues discovered that YTHDF2 stabilizes Tal1 mRNA and intensifies its expansion in HSCs (Li et al., 2018). Notably, Paris *et al.* demonstrated that YTHDF2 inhibition dramatically compromises the development and propagation of LSC. YTHDF2 decreased the m^6^A RNA stability of TNFR2, which is encoded by the Tnfrsf1b gene. Thus, loss of YTHDF2 caused AML cells to be more sensitive to TNF-induced apoptosis (Paris et al., 2019). Furthermore, repression of YTHDF2 increased global m^6^A methylation levels, decreased Tnfrsf1b mRNA and protein expression levels and substantially suppressed the t(8; 21) AML cell proliferation (Chen et al., 2021a). According to the recent research conducted by Sheng and others, YTHDC1 is highly expressed in AML and regulates leukemogenesis by MCM4, which is a critical regulator of DNA replication (Sheng et al., 2021). In another recent study, the data suggested that YTHDC1 is essential for AML cell survival, differentiation, and leukemogenesis. Mechanically, YTHDC1 undergoes liquid–liquid phase separation by binding to m^6^A to form dynamic nuclear condensates. YTHDC1 depletion leads to increased colocalization of MYC mRNA with PAXT components which mediated nuclear m^6^A mRNA decay (Cheng et al., 2021a). In addition, IGF2BP1 directly binds to ALDH1A1, HOXB4 and MYB mRNAs and elevates the expressions of these targets in AML cells (Elcheva et al., 2020).

In general, changes in m^6^A modification levels on PTEN, MYC, MYB, ASB2, RARA, CEBPA, and PFKP eventually contribute to the occurrence of AML.

### Lung Cancer

According to Global Cancer Statistics 2020, lung cancer (LC) is currently one of the most prevalent lethal malignancies and the leading cause of cancer-related deaths throughout the world (Sung et al., 2021). The TCGA and GTEx datasets indicate that expression levels of m^6^A regulators including METTL3, RBM15, HNRNPC, and KIAA1429 were correlated with the overall survival of LUAD patients (Wang et al., 2021a). Furthermore, METTL3, YTHDF1/2, RBM15, HNRNPC, and KIAA1429 expression levels were up-regulated, whereas METTL14, FTO, WTAP, ZC3H13, and YTHDC1 expression levels were down-regulated in LUAD (Li et al., 2020b). In NSCLC tissue and cells, METTL3 and its target oncogenes Bcl-2, EZH2, and JUNB, are up-regulated, correlating with LC progression status (Wanna-Udom et al., 2020; Zhang et al., 2021a). However, Wu *et al.* indicated that the expression of METTL3 is down-regulated in human LUAD tissues. METTL3, acting as an anti-oncogene, maintains FBXW7 translation and expression through an m^6^A-dependent mechanism in LUAD (Wu et al., 2021a).

At the same time, m^6^A demethylase FTO is identified as a prognostic factor in LUSC. It was found that FTO increases the MZF1 expression levels by decreasing its mRNA stability, therefore contributing to pro-tumorigenic effects on the cell behavior of LUSC (Liu et al., 2018). Consistently, a recent research by Li *et al.* also observed that silencing FTO represses the growth of NSCLC cells by reducing the expression level of USP7 (Li et al., 2019a). Recently, it was reported that FTO inhibition in NSCLC cells decreases E2F1 expression level by regulating m^6^A modification of E2F1. In the *in vivo* and *in vitro* experiments, FTO/E2F1/NELL2 axis was proposed to be responsible for augmenting NSCLC cell migration, invasion, and metastasis (Wang et al., 2021b). Meanwhile, the importance of mRNA methylation erased by ALKBH5 in LC cells is an emerging research subject. For instance, ALKBH5 can repress the tumor growth and metastasis of NSCLC by reducing the YAP activity, indicating its potential treatment value for LC (Jin et al., 2020). However, several controversial reports demonstrated that ALKBH5 functions as an oncogene in the progress of LC patients and cells. Zhu *et al.* revealed that ALKBH5 promotes the malignant biological properties of NSCLC by decreasing the TIMP3 mRNA stability and protein expression (Zhu et al., 2020). ALKBH5 overexpression could distinctly accelerate the expression and stability of m^6^A target oncogenes (SAMD7, SOX2, and MYC) in the YTHDF2-dependent pathway, thereby resulting in aggressive phenotypes of KRAS-mutated LC (Zhang et al., 2021b).

A recent study from a metabolic perspective indicated that YTHDF2 directly binds to the m^6^A modification site of 3′-UTR of 6PGD to promote 6PGD mRNA translation but does not cause 6PGD transcription degradation (Sheng et al., 2020). YTHDC2 was shown to destabilize SLC7A11 mRNA by its m^6^A-reading YTH domain. What’s more, METTL3-guided m^6^A methylation of SLC7A11 mRNA at its 3′UTR region is required for YTHDC2 to suppress the antioxidant function of LUAD cells by accelerating SLC7A11 mRNA decay (Ma et al., 2021a). In addition to SLC7A11, SLC3A2 was considered important for YTHDC2-induced ferroptosis in LUAD cells. Further investigation pointed out that HOXA13 accelerates SLC3A2 transcription, and YTHDC2 destabilizes HOXA13 mRNA *via* its YTH m^6^A-recognizing domain (Ma et al., 2021b).

In summary, the aforementioned research studies illustrated that m^6^A patterns in RNA participate in lung tumor biology and that m^6^A modifications might point to a potential therapeutic target for LC treatment.

### Hepatocellular Carcinoma

Hepatocellular carcinoma (HCC) is a primary liver malignancy with poor long-term prognosis and high mortality, accounting for over 80% of primary liver cancers (Bray et al., 2018). METTL3 expression has been observed to be associated with poor prognosis in HCC patients. It has been reported that the high expression of METTL3 in HCC leads to higher m^6^A methylation levels of SOCS2 and decreases SOCS2 mRNA expression by degrading SOCS2 mRNA transcripts through a YTHDF2-dependent pathway (Chen et al., 2018). Overexpression of METTL14 significantly increases the USP48 mRNA stability and expression levels in Huh-7 and HepG2 cells, thereby mediating SIRT6 ubiquitination and glycolysis (Du et al., 2021). A new study has revealed that depletion of METTL16 remarkably inhibits the growth, migration, and invasion of HCC cells and suppresses tumor growth *in vivo*. METTL16 facilitates translation initiation through interactions with eIF3a/b. Thus, targeting the METTL16-eIF3a/b axis represents a new therapeutic strategy for HCC (Su et al., 2022). Chen et al. found that silencing of WTAP greatly prolongs the half-life of ETS1 mRNA and reinforce the expression level of ETS1 mRNA by an m^6^A-HuR-dependent pathway (Chen et al., 2019).

In the diethylnitrosamine-induced HCC mice, hepatic FTO deficiency (FTO^L-KO^) not only increased tumor numbers but also increased numbers of larger tumors, revealing the protective role of FTO in the development of HCC *in vivo*. It showed that CUL4A protein expression was induced in FTO^L-KO^ livers (Mittenbühler et al., 2020). However, another contradictory study signified that the highly expressed FTO was related to the poor prognosis of HCC patients. Knockdown of FTO could decrease PKM2 to regulate the HCC progression (Li et al., 2019b). Coordinately, ALKBH5-mediated m^6^A demethylation results in a post-transcriptional inhibition of LYPD1, and LYPD1 could be recognized and stabilized by the m^6^A effector IGF2BP1 (Chen et al., 2020b). A recent inverse study revealed that ALKBH5 is overexpressed and predicts poor prognosis in HBV-HCC patients. The ectopic high expression level of ALKBH5 is induced by HBx-mediated H3K4me3 modification of ALKBH5 gene promoter in a WDR5-dependent manner. Also, ALKBH5 stabilizes HBx mRNA by decreasing m^6^A modification, therefore composing a positive HBx-WDR5-H3K4me3 feedback loop (Qu et al., 2021).

Silencing YTHDF2 might inhibit the liver CSC phenotype and cancer metastasis by modulating the m^6^A levels in the 5′-UTR of OCT4 mRNA (Zhang et al., 2020a). Furthermore, it revealed that m^6^A-binding protein (IGF2BP1, IGF2BP2, or HNRNPC) is statistically significantly up-regulated in tumor tissues of liver cancer, showing that it might be an independent prognostic factor (Müller et al., 2019; Pu et al., 2020). Functional experiments showed that loss of IGF2BP2 reduces HCC proliferation and tumor growth. Mechanistically, IGF2BP2 could direct recognize and bound to the FEN1 mRNA m^6^A site and enhance its stability (Pu et al., 2020).

These articles strongly suggest that abnormal m^6^A modification plays a crucial role in the occurrence and development of HCC, represents a promising diagnosis and prognosis biomarker and regards as an effective therapeutic target in HCC patients.

### Glioblastoma

Glioblastoma (GBM) is an aggressive adult malignant brain tumor. Despite recent advancements in surgery, radiation therapy, and chemotherapy, the median survival of glioma patients is less than 14 months after diagnosis (Uddin et al., 2020). The lack of success for GBM treatment is tumor heterogeneity, among which a population entity is identified as glioblastoma stem cells (GSCs). The presence of these GSCs elicits self-renew, renders GBM treatment-resistance for conventional therapy, and contributes to recurrence by sustaining long-term tumor growth (Mitchell et al., 2021). Hence, studying the new therapies that target GSCs are urgently needed. Cui et al. first reported that METTL3/14 dramatically inhibit GSC proliferation, self-renewal ability, and tumorigenesis by modulating ADAM19 (Cui et al., 2017). Li *et al.* further determined that decreased METTL3 expression but increased FTO expression was contributed to a reduced m^6^A level in RNA in glioma tissues and U251 cells (Li et al., 2019c). In contrast, in another publication, it has been shown that METTL3 as an oncogene is clearly more abundant in gliomas. Further analysis points out that METTL3 stabilizes SOX2 mRNA through binding and methylating specific adenines in the SOX2-3′UTR (Visvanathan et al., 2018). Li *et al.* also indicated that elevated expression of METTL3 is associated with aggressiveness of malignant gliomas. Interference of METTL3 but not METTL14 suppresses the self-renewal, proliferation, and growth of GSCs. Integrated transcriptome and m^6^A-IP-seq analyses uncovered that altered expression level of METTL3 targets splicing factors SRSF3, SRSF6, and SRSF11 by decreasing its m^6^A modification levels, thus resulting in YTHDC1-dependent nonsense-mediated mRNA decay of SRSFs mRNA transcripts and decreased protein expression of SRSFs (Li et al., 2019d). An added value of Tassinari’s work is that METTL3 main targets ADAR1 and eventually leads to modulating cell proliferation and tumor growth. Silencing METTL3 or YTHDF1 significantly decreases the ADAR1 protein level, indicating that METTL3-mediated m^6^A modification regulates ADAR1 protein expression by YTHDF1-dependent post-transcription of ADAR1 (Tassinari et al., 2021).

In GSCs, m^6^A demethylase ALKBH5 has been shown to be highly expressed and binds to the FOXM1 directly. In this process, siRNA against ALKBH5 contributes to a decrease in FOXM1 nascent transcripts but not FOXM1 RNA and then alters the expression of FOXM1 mature RNA or protein (Zhang et al., 2017). Recent reports suggest that m^6^A reader YTHDF2 promotes cell growth of GSCs by promoting MYC stability (Dixit et al., 2021).

Collectively, these findings open up avenues for providing new therapeutic opportunities in glioma treatment.

### Breast Cancer

Breast cancer (BC) continues to be the second leading cause of cancer-related deaths among women worldwide (Loibl et al., 2021). The mortality from BC was primarily due to metastasis and chemo-resistance (Garcia-Martinez et al., 2021). Recent studies have investigated m^6^A-related mechanisms in BC, thereby providing new therapeutic approaches for the BC treatment. In BC, METTL3 was reported to be frequently elevated, implying an oncogene role. METTL3 promotes the HBXIP mRNA methylation and its expression. Interestingly, HBXIP also facilitates METTL3 expression by restraining tumor suppressor miRNA let-7g, which stimulates METTL3 expression through targeting its 3′UTR, thereby forming a positive feedback loop of HBXIP/let-7g/METTL3/HBXIP (Cai et al., 2018). Another report indicated that METTL3 promotes cell proliferation and inhibits cell apoptosis by targeting Bcl-2 in BC (Wang et al., 2020c). However, METTL3 was found to be a tumor suppressor in triple-negative breast cancer (TNBC). It suppressed TNBC cell migration, invasion, and adhesion by decreasing the COL3A1 expression (Shi et al., 2020a). METTL14 was recognized and recruited by elevating LNC942, which in turn increased METTL14-dependent m^6^A methylation expression levels and its associated mRNA stability and protein expression of downstream targets CXCR4 and CYP1B1 in BC (Sun et al., 2020).

In addition, the expression of FTO is higher in BC clinical samples and MDA-MB-231, MCF-7, and 4T1 cell lines. Blockade of FTO could induce BNIP3 methylation and reduce BNIP3 degradation, therefore alleviating BC cell proliferation, colony formation, and metastasis (Niu et al., 2019). Under hypoxic conditions, HIF-1α and HIF-2α stimulate ALKBH5 expression, which decreases m^6^A demethylation and NANOG mRNA stability in breast cancer stem cells (BCSCs). Elevated NANOG accelerates the enrichment of BCSCs (Zhang et al., 2016a).

### Cervical Cancer and Ovarian Cancer

The development of transcriptome sequencing provides a new approach for the discovery and therapy of cervical cancer (CC) and ovarian cancer (OC). The high expression level of METTL3 in the CC was significantly associated with poor disease-free survival and overall survival (Ni et al., 2020). Wang *et al.* found that METTL3 targets the 3′-UTR of HK2 mRNA and recruits YTHDF1 to enhance HK2 stability, thereby promoting the Warburg effect and the proliferation of CC (Wang et al., 2020d). Furthermore, Hu *et al.* suggested that METTL3 increases the RAB2B expression and RAB2B mRNA stability *via* an IGF2BP3-dependent pathway (Hu et al., 2020). However, Yang *et al.* showed that METTL3 can increase the m^6^A level of ZFAS1 but cannot influence its expression (Yang et al., 2020c). FTO serves as an oncogenic regulator in the proliferation and migration of CC, resulting in higher levels of m^6^A modification in E2F1 and Myc transcripts, which causes increased expression of E2F1 and Myc (Zou et al., 2020). In the recent study by Wang *et al.*, depletion of YTHDF1 remarkably inhibits CC cell proliferation, migration, and invasion and induces apoptosis. Using the online meRIP-seq, meRIP-seq, and Ribo-seq data analysis upon YTHDF1 knockdown, it was revealed that YTHDF1 directly targets RANBP2. Further investigation found that YTHDF1 regulates RANBP2 protein expression in an m^6^A-dependent manner (Wang et al., 2021c).

In OC tissues, METTL3 promotes the AXL translation independent of its catalytic activity (Hua et al., 2018). In endometrioid epithelial OC, knockdown of METTL3 decreases the m^6^A level, whereas knockdown of METTL14 or WTAP has no influence (Ma et al., 2020). Several studies have described the role of YTHDF1/2 in OC progressions. For instance, YTHDF1 interacts with the EIF3C mRNA and promotes EIF3C protein expression and the overall translational output in OC (Liu et al., 2020a). Knockdown of YTHDF2 using specific shRNAs significantly increases BMF mRNA expression and prolongs its half-life in OC (Xu et al., 2021).

As mentioned previously, m^6^A editing is intimately involved in the phenotype and mechanism of tumorigenesis, suggesting the possibility of m^6^A-targeted therapies in CC and OC.

## M^6^A Regulators-Modified Noncoding RNA in Cancers

**FIGURE 2:**
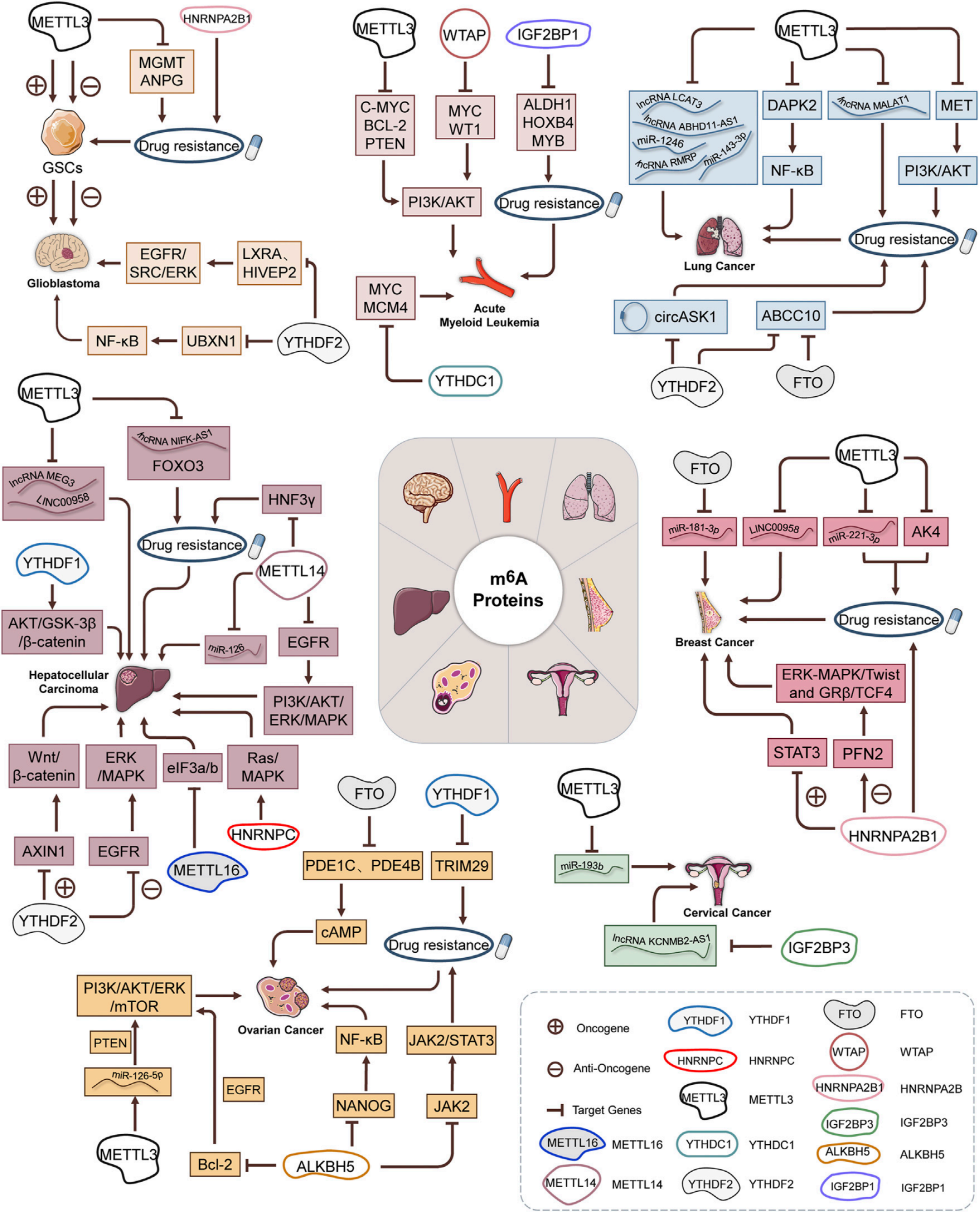
M^6^A RNA methylation steers RNA regulation in cancer progression.

An increasing number of studies have explored the control of ncRNAs (lncRNA, miRNA, and circRNA, *etc*.) transport, stability, degradation processes, and expression modified by m^6^A regulators (Figure 2 and Table 2). LCAT3 is a novel lncRNA, and its stability is regulated by METTL3. It was revealed that altering the m^6^A modification level of LCAT3 can significantly affect its binding with FUBP1 and regulate c-MYC expression, thereby influencing the proliferation and survival of LUAD (Qian et al., 2021). Similarly, Xue *et al.* have found that lncRNA ABHD11-AS1 indicates an unfavorable prognosis of NSCLC patients and promotes NSCLC proliferation. METTL3 accelerates the m^6^A and ABHD11-AS1 transcript stability to increase its expression. Furthermore, ABHD11-AS1/EZH2/KLF4 axis exerts the regulative role on the Warburg effect of NSCLC (Xue et al., 2021). A lipogenesis-related lncRNA, LINC00958, showed to aggravate HCC growth and progression *in vitro* and *in vivo*. METTL3-mediated m^6^A modification resulted in LINC00958 up-regulation by stabilizing its RNA transcript, which subsequently facilitates lipogenesis through the miR-3619-5p/HDGF axis (Zuo et al., 2020). Two other recent studies reported a similar phenotype and confirmed that METTL3 is critical for maintaining the malignant phenotypes by targeting lncRNA MEG3/miR-544b/BTG2 and lncRNA NIFK-AS1/miR-637/AKT1 of HCC cells (Chen et al., 2021b; Wu et al., 2021b). Similarly, high expression of METTL3-mediated m^6^A modification could promote BC tumorigenesis by up-regulating RNA transcript stability and expression levels of its target gene LINC00958 (Rong et al., 2021). It was intriguing that in the established BC lung metastasis BC^LMF3^cells, METTL3 is increased, but FTO is decreased. *In vivo* and clinical studies indicated that METTL3 methylates long non-coding RNA KRT7-AS at 877 A (with GGAC motif) and increases the stability of a KRT7-AS/KRT7 mRNA duplex by binding with IGF2BP1/HuR complexes. In addition, YTHDF1/eEF-1 is responsible for FTO-regulated translational elongation of KRT7 mRNA, with methylated A950 in KRT7 exon 6 as the key site for methylation. Thus, all these data confirmed that m^6^A promotes BC lung metastasis by regulating the KRT7/KRT7-AS axis (Chen et al., 2021c). Yet, Yu *et al.* presented the regulatory role of ALKBH5 in lncRNA methylation. It was demonstrated that ALKBH5 demethylates lncRNA RMRP and leads to the increase of lncRNA RMRP expression. ALKBH5 silence compromises LUAD development and propagation *in vitro* and *vivo*, which is partially reversed by RMRP (Yu and Zhang, 2021). Notably, a novel lncRNA FGF13-AS1 destabilized Myc mRNA through binding IGF2BPs and disrupted the interaction between Myc mRNA and IGF2BPs (Ma et al., 2019). Furthermore, IGF2BP3 stabilizes and interacts with lncRNA KCNMB2-AS1 by three m^6^A modification motifs (TGGAC) on KCNMB2-AS1 in CC (Zhang et al., 2020b).

**TABLE 2:** M^6^A regulators-modified noncoding RNA in various cancers.

M^6^A regulator	Cancer type	Target ncRNA	Function	Regulation	Mechanism of m6A recognition	Reference
METTL3	LC	lncRNA LCAT3	Oncogene	Up-regulation	Stabilize LCAT3 mRNA, increase LCAT3 expression, and promote proliferation, survival, migration/invasion, and metastasis of LUAD	34274028
METTL3	LC	lncRNA ABHD11-AS1	Oncogene	Up-regulation	Stabilize ABHD11-AS1, increase ABHD11-AS1 expression, and promote proliferation	32892348
METTL3	HCC	LINC00958	Oncogene	Up-regulation	Stabilize LINC00958, increase LINC00958 expression, and promote lipogenesis, proliferation, migration, invasion, and cell growth	31915027
METTL3	HCC	lncRNA MEG3	Oncogene	Down-regulation	Destabilize MEG3, decrease MEG3 expression, and promote proliferation, migration, and invasion	34163177
METTL3	BC	LINC00958	Oncogene	Up-regulation	Stabilize LINC00958, increase LINC00958 expression, promote cell proliferation and tumor growth, and inhibit apoptosis	33531456
ALKBH5	LC	lncRNA RMRP	Oncogene	Up-regulation	Increase RMRP expression and promote tumorigenesis	33934179
IGF2BP3	CC	lncRNA KCNMB2-AS1	Oncogene	Up-regulation	Stabilize KCNMB2-AS1, increase KCNMB2-AS1 expression, and promote tumorigenesis	33028109
METTL3	LC	miR-1246	Oncogene	Up-regulation	Increase pri-miR-1246 and PEG3 expressions and promote cell growth, survival, and invasion	33898106
METTL3	LC	miR-143-3p	Oncogene	Up-regulation	Increase pri-miR-143-3p expression	31823788
METTL3	CC	miR-193b	Oncogene	Down-regulation	Increase miR-193b expression and decrease pri-miR-193b expression	34178650
METTL3	OC	miR-126-5p	Oncogene	Up-regulation	Increase mature miR-126-5p and promote cell development and tumorigenesis	32939058
METTL14	HCC	miR-126	Anti-oncogene	Down-regulation	Decrease mature miR-126 and inhibit tumor invasion and metastasis	27774652
FTO	BC	miR-181b-3p	Oncogene	Down-regulation	Decrease miR-181b-3p expression and promote cell migration and invasion	32805088

LIN28B-AS1 and circXPO1 were recently reported to promote the LUAD cell progression by interacting with IGF2BP1. It displayed that LIN28B-AS1 inhibits the LIN28B mRNA stability *via* suppressing IGF2BP1 and then promotes LUAD cell proliferation and metastasis (Wang et al., 2019a). In another study, circXPO1 enhances LUAD progression by the circXPO1/IGF2BP1-CTNNB1 axis (Huang et al., 2020a). According to Ji *et al.*, a novel m^6^A-modified circRNA circARHGAP12 could bind to IGF2BP2 to increase the stability of FOXM1 mRNA, forming the circARHGAP12/IGF2BP2/FOXM1 complex, therefore accelerating the proliferation and migration of CC cells (Ji et al., 2021).

Interesting, Li indicates the interaction between miR-590-5p and circPUM1 or METTL3 in A549 and H1650 cells. MiR-590-5p can inhibit cell growth and glycolysis by directly targeting METTL3, and circPUM1 indirectly regulate METTL3 *via* miR-590-5p. Ultimately, the study revealed that circPUM1 facilitates NSCLC tumorigenesis by targeting the miR-590-5p/METTL3 axis (Li et al., 2021a). Using the TCGA and GEO database, Chi et al. constructed the regulatory network of circRNA-miRNA-m^6^A RNA methylation. Hsa_circ_0007456 (circMAP2K4) suggested acting as an hsa-miR-139-5p sponge to promote the expression and activity of YTHDF1 (Chi et al., 2021).

On the other hand, inhibition of METTL3 decreases miR-1246/miR-143-3p but increases pri-miR-1246/miR-143-3p, suggesting that METTL3 could promote the transition of precursor-miRNA to mature miRNA (Wang et al., 2019b; Huang et al., 2021b). In the work of Huang *et al.*, blockade of METTL3 in CC decreases the miR-193b expression and increases pri-miR-193b expression. The subsequent evidence proves that METTL3 modulates miR-193b mature process by promoting pri-miR-193b m^6^A methylation level (Huang et al., 2021c). A new study has revealed that METTL3 increases mature miR-126-5p by the m^6^A modification of pri-miR-126-5p in OC (Bi et al., 2021). In addition, METTL14 interacts with the microprocessor protein DGCR8 to modulate the pri-miR-126 process and suppresses the HCC carcinogenesis (Ma et al., 2017). In another recent study, the overexpressed FTO was more confirmed in HER2-positive BC patients and cells. FTO targets miR-181b-3p/ARL5B axis to promote cell migration and invasion (Xu et al., 2020).

## The Signaling Pathway Involved in m^6^A RNA Methylation

A study reported by Ly P Vu et al. elucidated that METTL3 induces m^6^A methylation levels of its target genes such as c-MYC, BCL2 and PTEN in AML MOLM-13 cells, thus promoting these oncogenes’ translation. Consequently, loss of METTL3 induces cell differentiation and apoptosis in MOLM-13 cells by the PI3K/AKT pathway and delays leukemia progression in mice *in vivo* (Vu et al., 2017). In NSCLC, increasing levels of METTL3 significantly down-regulates DAPK2 mRNA and protein expressions and its mRNA stability by activating the NF-κB pathway, thus contributing to the NSCLC tumorigenesis (Jin et al., 2021). In OC cells, METTL3 deficiency alleviated the progression and tumorigenesis by inhibiting the miR-126-5p expression *via* suppressing the PTEN-medicated PI3K/Akt/mTOR pathway (Bi et al., 2021). Shi *et al.* indicated that decreased METTL14 expression reduces m^6^A modification levels but augment the mRNA and protein expression levels of EGFR. In addition, METTL14 can inhibit cell migration, invasion, and EMT *via* targeting the EGFR/PI3K/AKT signaling pathway in HepG2 and MHCC-LM3 cells (Shi et al., 2020b).

Recently, Naren *et al.* demonstrated that high WTAP expression was linked with higher peripheral WBC and higher peripheral BLAST% in AML. WTAP mainly regulated proteins downstream of the PI3K/AKT signaling pathway, thus affecting the RNA stability and expressions of MYC mRNA through mRNA m^6^A methylation (Naren et al., 2021).

In the high-grade serous OC, FTO expression is down-regulated and inhibits cell proliferation/self-renewal and suppresses ovarian carcinogenesis. FTO mediates m^6^A demethylation in the 3′UTR of PDE4B and PDE1C mRNA and reduces the mRNA stability through second messenger 3′, 5′-cAMP signaling (Huang et al., 2020b). A research conducted by Zhu *et al.* suggested that ALKBH5 enhances cellular proliferation and migration, inhibits autophagy through activating the EGFR-PIK3CA-AKT-mTOR signaling pathway, facilitates the BCL-2 mRNA demethylation and stabilization, and promotes the interaction between BCL-2 and BECN1 (Zhu et al., 2019b). Jiang and others have found that in OC tissues and cells, ALKBH5 targets NANOG and promotes OC development through stimulating the NF-κB pathway (Jiang et al., 2020).

Lately, evidence thus far indicated that YTHDF1 deficiency inhibits the EMT process and AKT/GSK-3β/β-catenin signaling pathway in HCC (Bian et al., 2020). As elucidated by Li *et al.*, YTHDF2 deficiency significantly inhibited LUAD tumorigenesis. It controls the LUAD cell proliferation, colony formation, and migration by targeting the AXIN1/Wnt/β-catenin signaling pathway (Li et al., 2021b). Recently, Zhang *et al.* proposed the opposite results that YTHDF2 is down-regulated, which served as a tumor suppressor in four HCC cell lines under hypoxia. Consistently, the decreased YTHDF2 protein catalyzes the m^6^A methylation of EGFR mRNA by stabilizing and favoring a higher EGFR mRNA and protein expression levels, which, in turn, impairs the ERK/MAPK pathway (Zhong et al., 2019). In GBM cells, YTHDF2 can mediate m^6^A dependent mRNA decay to inhibit the mRNA and protein expressions of LXRA and HIVEP2 under the activation of EGFR/SRC/ERK signaling. This effect is involved in GBM tumorigenesis by enhancing cholesterol dysregulation (Fang et al., 2021). Next, another new study found that transfected with YTHDF2 specific shRNA significantly increases the levels of mRNA and protein of UBXN1. Meanwhile, YTHDF2 accelerates UBXN1 mRNA degradation in GBM by recognizing the m^6^A modification mediated by METTL3, which, in turn, activates NF-κB (Chai et al., 2021).

Furthermore, HNRNPA2B1 was shown to interact with STAT3 and stimulate the activation of the STAT3 signaling pathway. HNRNPA2B1 knockout increases BC cell apoptosis, alleviates autophagy, and declines tumor growth *in vitro* and *in vivo* (Gao et al., 2021a). In contrast to the cancer research findings, knockout of HNRNPA2B1 by CRISPR/CAS9 method promotes the TNBC cell migration and invasion but alleviates tumor growth by activating the ERK-MAPK/Twist and GR-beta/TCF4 signaling pathways. HNRNPA2B1 binds directly to the PFN2 mRNA at the site of the UAGGG sequence of the 3′-UTR and reduces its stability (Liu et al., 2020b). Specifically, another report has indicated that decreased HNRNPC expression reduces the activation of the Ras/MAPK signaling pathway (Hu et al., 2021) (Figure 2 and Table 3).

**TABLE 3:** Signaling pathways involved in m^6^A RNA methylation.

M^6^A regulator	Cancer type	Target gene	Function	Regulation	Mechanism of m6A recognition	Related signaling pathway	Reference
METTL3	AML	c-MYC, BCL2, and PTEN	Oncogene	Up-regulation	Promote translation of c-MYC, BCL2, and PTEN and inhibit cell differentiation and apoptosis	PI3K/AKT	28920958
METTL3	LC	DAPK2	Oncogene	Down-regulation	Destabilize DAPK2 mRNA, decrease DAPK2 expression, and promote cell proliferation and migration abilities	NF-κB	34298122
METTL3	OC	miR-126-5p	Oncogene	Up-regulation	Increase mature miR-126-5p and promote cell development and tumorigenesis	PTEN/PI3K/Akt/mTOR	32939058
METTL14	HCC	EGFR	Anti-oncogene	Up-regulation	Stabilize EGFR mRNA, increase p-EGFR expression, and inhibit cell migration, invasion, and EMT	PI3K/AKT/ERK/MAPK	33380825
WTAP	AML	MYC and WT1	Oncogene	Down-regulation	Destabilize MYC mRNA, decrease MYC expressions, and promote cell proliferation, tumorigenesis, cell cycle, and differentiation	PI3K/AKT	32880751
FTO	OC	PDE1C and PDE4B	Anti-oncogene	Up-regulation	Destabilize PDE1C/PDE4B, increase PDE1C/PDE4B mRNA expressions, and inhibit cell proliferation/self-renewal and tumorigenesis	cAMP	32606006
ALKBH5	OC	BCL-2	Oncogene	Up-regulation	Stabilize BCL-2, increase BCL-2 expression, promote cellular proliferation and migration, and inhibit autophagy	EGFR/PIK3CA/AKT/mTOR	30987661
ALKBH5	OC	NANOG	Oncogene	Up-regulation	Promote cell proliferation, migration, invasion, and tumor growth and inhibit apoptosis	NF-κB	32329191
YTHDF1	HCC	—	Oncogene	—	Promote cell proliferation, migration, and invasion and inhibit apoptosis	AKT/GSK-3β/β-catenin	33363211
YTHDF2	HCC	AXIN1	Oncogene	Down-regulation	Destabilize AXIN1 mRNA, decrease AXIN1 expression, and promote cell proliferation and migration	Wnt/β-catenin	33980824
YTHDF2	HCC	EGFR	Anti-oncogene	Up-regulation	Stabilize EGFR mRNA, increase EGFR expression, and inhibit cell proliferation and tumor growth	ERK/MAPK	30423408
YTHDF2	GBM	LXRA and HIVEP2	Oncogene	Down-regulation	Destabilize LXRA and HIVEP2 mRNA, decrease LXRA and HIVEP2 expressions, and promote cholesterol dysregulation, cell proliferation, invasion, and tumorigenesis	EGFR/SRC/ERK	33420027
YTHDF2	GBM	UBXN1	Oncogene	Down-regulation	Destabilize UBXN1 mRNA, decrease UBXN1 expression, and promote cell proliferation and migration	NF-κB	34246306
HNRNPA2B1	BC	STAT3	Oncogene	—	Increase p‐STAT3 expression and promote cell proliferation and tumor growth	STAT3	33399232
HNRNPA2B1	BC	PFN2	Anti-oncogene	Up-regulation	Destabilize PFN2 mRNA, increase PFN2 expressions, and suppress cell migration and invasion	ERK-MAPK/twist and GR-beta/TCF4	31901866
HNRNPC	HCC	—	Oncogene	—	Promote cell proliferation, migration, and invasion	Ras/MAPK	33937074

## The Influence of m^6^A RNA Methylation on Drug Resistance

Gefitinib resistance is also shown as a major obstacle to the successful therapy of NSCLC. A recent study revealed that METTL3 is up-regulated in gefitinib resistant LUAD tissues. Knocking down METTL3 leads to the lower expression of the MET and PI3K/AKT signaling pathway, which induces the sensitivity of PC9 and H3255 cells to gefitinib (Gao et al., 2021b). Using exosomal RNA-seq, Xiao et al. first found that FTO interference not only increased the gefitinib-resistant PC9/GR cells to gefitinib but also decreased the acquired resistance of gefitinib-sensitive PC9 cells in exosomes. The FTO/YTHDF2/ABCC10 axis was involved in the intercellular transmission of gefitinib-resistant cell-derived exosomal-FTO-mediated gefitinib resistance (Xiao et al., 2021). Moreover, Wang et al. identified that increased YTHDF2-mediated endoribonucleolytic cleavage of m^6^A-modified circASK1 contributes to down-regulation of circASK1 expression, which induces gefitinib-resistance in LUAD cells *in vitro* (Wang et al., 2021d).

Sorafenib is the first FDA approved targeted agent for advanced HCC but only exhibits notable therapeutic effects for a minority of HCC patients. As Chen *et al.* suggested, METTL3-mediated NIFK-AS1 down-regulation functions to increase the uptake of sorafenib, thereby enhancing sorafenib resistance of HCC (Chen et al., 2021b). Lin *et al.* further confirmed the role of METTL3 in the resistance of HCC to sorafenib therapy. On the contrary, METTL3 deficiency evidently improved autophagy-induced sorafenib resistance by METTL3/FOXO3 axis (Lin et al., 2020). Subsequently, another analogous study demonstrated that there is a remarkable correlation between HNF3γ expression and the levels of METTL14 but not METTL3, WTAP, or FTO in 57 patient HCCs. METTL14 knockdown apparently decreases HNF3γ mRNA stability of HCC cells. Furthermore, enforced HNF3γ expression enhances the sorafenib sensitivity and promotes the differentiation of HCC cells and liver cancer stem cells (CSCs) (Zhou et al., 2020b).

Notably, a recent study has shown that abnormal METTL3 expression plays a pivotal role in regulating temozolomide (TMZ) resistance in parental-sensitive and resistant GBM cell lines. Repression of METTL3 induces the TMZ-sensitivity of GBM cells *in vitro* and *in vivo* by decreasing the MGMT and ANPG expression in an m^6^A dependent manner (Shi et al., 2021b). Deng et al. performed an observational study investigating the effect of HNRNPA2/B1 in GBM tumorigenesis and chemoresistance for TMZ. HNRNPA2/B1 down-regulating inhibits p-STAT3 and MMP-2 levels and reduces GBM cell viability, adhesion, migration, invasion, and chemoresistance for TMZ capacity (Deng et al., 2016).

In cisplatin (DDP)-resistant LC cells, METTL3/YTHDF3 complex promotes the level of m^6^A modification of lncRNA MALAT1 and its stability. The METTL3-MALAT1-miR-1914-3p-YAP axis could induce the DDP resistance, growth, and metastasis (Jin et al., 2019). Furthermore, ALKBH5 is up-regulated in DDP-resistant epithelial OC, thus accelerating cell DDP resistance both *in vivo* and *in vitro*. ALKBH5 formed a loop with HOXA10 that activates the JAK2/STAT3 pathway through mediating JAK2 mRNA m^6^A demethylation and concomitantly promoting epithelial OC cell resistance to DDP (Nie et al., 2021). Subsequently, YTHDF1 augments the translation of TRIM29 in an m^6^A-dependent manner by binding to TRIM29 mRNA, which was responsible for regulating the CSC-like characteristics of the DDP-resistant OC (Hao et al., 2021).

METTL3 high expression is associated with the high expression of AK4, thus contributing to tamoxifen (TAM) resistance in BC. siRNA-mediated knockdown of METTL3 in TAM-resistant MCF-7 cells significantly decreases AK4 protein levels, thereby resulting in inducing mitochondrial apoptosis and reducing ROS production (Liu et al., 2020c). A recent research conducted by Petri *et al.,* which focused on endocrine resistance, suggested that HNRNPA2B1 is overexpression in primary breast tumors. Suppression of HNRNPA2B1 significantly increases TAM and fulvestrant endocrine sensitivity in TAM-resistant LCC9 and LY2 cells (Petri et al., 2021). In Adriamycin (ADR)–resistant MCF-7/ADR cells augmented METTL3 increases the expression of miR-221-3p by enhancing pri-miR-221-3p maturation *via* accelerating m^6^A mRNA methylation. The functional axis of METTL3/miR-221-3p/HIPK2/Che-1 ultimately overcomes ADR resistance and reduces the side effects of chemotherapy in the treatment of BC (Pan et al., 2021).

In addition, WTAP promoted AML tumorigenesis and made AML cells resistant to chemotherapy drug daunorubicin (Naren et al., 2021). Knockdown of IGF2BP1 results in less colony-forming and higher drug sensitivity to chemotherapeutic drugs, including doxorubicin, cytarabine, and cyclophosphamide in AML cells (Figure 2 and Table 4) (Elcheva et al., 2020).

**TABLE 4:** Roles of m^6^A RNA methylation as regulators of drug resistance.

M^6^A regulator	Cancer type	Target gene	Function	Regulation	Influence on drug resistance	Reference
METTL3	LC	MET	Oncogene	Up-regulation	Induce gefitinib resistance	33491264
FTO/YTHDF2	LC	ABCC10	Oncogene	Down-regulation	Induce gefitinib resistance	33563765
YTHDF2	LC	circASK1	Oncogene	Down-regulation	Induce gefitinib resistance	34389432
METTL3	HCC	lncRNA NIFK-AS1	Oncogene	Up-regulation	Induce sorafenib resistance	34374933
METTL3	HCC	FOXO3	Anti-oncogene	Down-regulation	Inhibit sorafenib resistance	32368828
METTL14	HCC	HNF3γ	Anti-oncogene	Down-regulation	Inhibit sorafenib resistance	33361765
METTL3	GBM	MGMT and ANPG	Oncogene	Up-regulation	Induce temozolomide resistance	34336690
HNRNPA2B1	GBM	—	Oncogene	—	Induce temozolomide resistance	25586062
METTL3	LC	lncRNA MALAT1	Oncogene	Up-regulation	Induce cisplatin resistance	31818312
ALKBH5	OC	JAK2	Oncogene	Up-regulation	Induce cisplatin resistance	34496932
YTHDF1	OC	TRIM29	Oncogene	Up-regulation	Induce cisplatin resistance	33011193
METTL3	BC	AK4	Oncogene	Up-regulation	Induce tamoxifen resistance	32956623
HNRNPA2B1	BC	—	Oncogene	—	Induce tamoxifen and fulvestrant resistance	34273466
METTL3	BC	miR-221-3p	Oncogene	Up-regulation	Induce Adriamycin resistance	33420414
WTAP	AML	MYC and WT1	Oncogene	Down-regulation	Induce daunorubicin resistance	32880751
IGF2BP1	AML	ALDH1A1, HOXB4, and MYB	Oncogene	—	Induce doxorubicin, cytarabine, and cyclophosphamide resistance	31768017

## Novel Anticancer Agents Based on m^6^A RNA Methylation

M^6^A RNA methylation indicates new directions for therapeutic targets in cancer therapy and drug resistance. Therefore, inhibitors or regulators of m^6^A proteins may serve as potential therapeutics for the treatment of cancers, such as rhein, R-2HG, meclofenamic acid (MA), FB23, and MO-I-500. The first FTO inhibitor, rhein, a natural product, has been identified to effectively compete with m^6^A-containing RNA for competitively binding to the FTO catalytic domain (Chen et al., 2012). Nevertheless, rhein is not only an FTO-specific inhibitor but also an inhibitor of other ALKB family demethylases (Li et al., 2016). R-2HG is a competitive inhibitor of FTO. It directly binds to FTO protein, inhibits FTO activity, and sensitizes the cells to commonly used chemotherapy agents as well as exerts antileukemia effects through increasing global m^6^A modification levels in R-2HG-sensitive AML (Su et al., 2018). Another study has revealed that the R-2HG/FTO axis exhibits the glycolytic inhibitory function, suggesting that R-2HG and specific FTO inhibitors, alone or in combination with other anticancer agents, provide new treatment options for AML therapy by targeting tumor metabolism and epigenetic modulation (Qing et al., 2021). A nonsteroidal anti-inflammatory drug MA was identified as a highly selective inhibitor of FTO over ALKBH5 (Huang et al., 2015). As the ethyl ester novel derivative of MA, MA2 inhibits GSC growth and self-renewal and severely suppresses GSC-induced tumorigenesis (Cui et al., 2017). Furthermore, MA2 promotes the chemoradiotherapy sensitivity of CSCC (Zhou et al., 2018). It is worth noting that another two new MA-derived inhibitors, FB23 and FB23-2, show much stronger potential than MA in inhibiting FTO-mediated demethylation (Gao et al., 2021c). In addition, FB23-2 has a stronger potential in targeting FTO protease, impairing AML cell proliferation, and promoting cell apoptosis (Huang et al., 2019). Similarly, MO-I-500 shows a greater inhibitory effect than previously reported rhein. It has been reported that MO-I-500 could significantly inhibit tumorigenesis of BC cells (Singh et al., 2016). Most recently, based on the structural design and synthesis, Huff *et al.* found two new FTO inhibitors, namely, FTO-02 and FTO-04. FTO-04 obviously inhibits the proliferation of patient-derived GSC (Huff et al., 2021). Also, Su *et al.* discovered two small-molecule compounds, namely CS1 and CS2, which can effectively act against FTO demethylation. The effectiveness of CS1 and CS2 is at least ten times higher than previously described FTO inhibitors, including FB23-2 and MO-I-500 (Gao et al., 2021c). Interestingly, studies have shown that some natural products such as Saikosaponin D, kaempferol, and plumbagin could also significantly inhibit FTO demethylation activity. For instance, saikosaponin D displays antileukemic effects *in vitro* and *in vivo* by targeting FTO/m^6^A signaling (Sun et al., 2021). Targeting FTO could reduce immune checkpoint gene expression, especially LILRB4, consequently enhancing AML cell sensitivity to T cell cytotoxicity and overcoming the hypomethylating agent decitabine-induced immune evasion. Thus, combined FTO inhibition with hypomethylating agents may exert synergistic effects in AML treatment (Su et al., 2020). The combination of FTO inhibitors with nilotinib declines the TKI-resistant phenotype and alleviates the biological processes of AML cells (Yan et al., 2018). In addition, Yang *et al.* indicated that combined treatment with FTO inhibitors and anti-PD-1 blockers might decrease resistance to immunotherapy in melanoma (Yang et al., 2019). These emerging data and discoveries have revealed that FTO-selective/nonselective inhibitors alone or in combination with conventional therapeutic agents may exhibit tremendous therapeutic potential for cancer treatment.

Except FTO inhibitors, other m^6^A proteins inhibitors may also be the promising target for m^6^A-related human cancers. STM2457, a new highly potent and selective first-in-class catalytic inhibitor of METTL3, has been proven to reverse the AML phenotype and prolong cell survival in various AML mouse models (Yankova et al., 2021). Also, Cheng *et al.* suggested that metformin inhibits BC cell proliferation by down-regulating METTL3 (Cheng et al., 2021b). In another recent study, a compound MV1035, based on the imidazobenzoxazin-5-thione scaffold, targets ALKBH5 and decreases U87 GBM cell line migration and invasiveness (Malacrida et al., 2020).

Several upstream regulators of m^6^A proteins could also alter the total m^6^A level *via* regulating m^6^A proteins, developing a potential and advantageous avenue for treating various cancers (Barbieri et al., 2017). For example, METTL3 up-regulation by miR-338-5p involves the m^6^A modification of c-Myc. The miR-338/METTL3/cMyc regulatory axis influences the growth and migration of LC cells (Wu et al., 2021c). In addition, miR-4443 reverses the NSCLC resistance to DDP through the METTL3/FSP1-mediated ferroptosis pathway (Song et al., 2021). A hematopoietic transcription factor SPI1 has been shown to target METTL14 and therefore inhibits the development of malignant hematopoietic cells (Weng et al., 2018). As a member of the carbonic anhydrases, CA4 interacts with WTAP and induces WTAP protein degradation by polyubiquitination in colon cancer (Zhang et al., 2016b).

Collectively, these inhibitors will not only elaborate the function and mechanism of m^6^A RNA methylation in carcinogenesis but also provide novel therapeutic strategies for cancer patients.

## Future Prospect

Emerging research has revealed that m^6^A RNA methylation participates in the regulation of the cancer malignant phenotype and chemo-/radio-resistance by modulating the expression of different targets or pathways, primarily through its impact on mRNA stability and translation efficiency. With increasing studies on the mechanism of m^6^A modification in cancers, it was illustrated that m^6^A modification regulates related RNA levels in more diverse and complex circumstances. Mostly, the m^6^A modification level in RNA is closely associated with the expression of writing and erasing genes, but m^6^A readers that bind to the modification site exert a series of biological functions. Increasing evidences point toward the idea that m^6^A regulators, particularly writers and erasers, show the double-edged sword regulation in the progression of cancer and often outcomes seem similar. For example, METTL3 might conduct dual roles in both HCC and BC (Table 1). However, it is unclear how writer and eraser genes selectively serve their differing effects and how the activity and expression of readers are regulated in cancer cells. The mechanisms need to be further elucidated. Though some potent and selective m^6^A enzyme inhibitors have shown promising effects in the development of cancer, more effective drugs related to m^6^A by structural design and synthesis and novel therapeutic strategies are expected to be explored. In addition, the combinations of such m^6^A inhibitors and existing therapeutic agents could provide a new perspective approach in the treatment of cancers in the future.

## Abbreviations

ADR, Adriamycin; ALKBH5, ALKB homolog 5; AML, acute myeloid leukemia; BC, breast cancer; CC, cervical cancer; CSCC, cervical squamous cell carcinoma; CSCs, cancer stem cells; DDP, cisplatin; FTO, fat mass and obesity-associated protein; GBM, glioblastoma; GSCs, glioblastoma stem cells; HNRNPs, heterogeneous nuclear ribonucleoprotein family; HSCs, hematopoietic stem cells; HSPCs, hematopoietic stem progenitor cells; IGF2BPs, insulin-like growth factor 2 mRNA binding proteins; LC, lung cancer; LIHC, liver hepatocellular carcinoma; lncRNAs, long noncoding RNAs; LUAD, lung adenocarcinoma; MA, meclofenamic acid; METTL3, methyltransferase-like 3; miRNAs, microRNAs; ncRNAs, noncoding RNAs; NSCLC, non-small cell lung cancer; OC, ovarian cancer; R-2HG, R-2-hydroxyglutarate; TAM, tamoxifen; TMZ, temozolomide; TNBC, triple-negative breast cancer; WTAP, Wilms’ tumor 1-associated protein.
